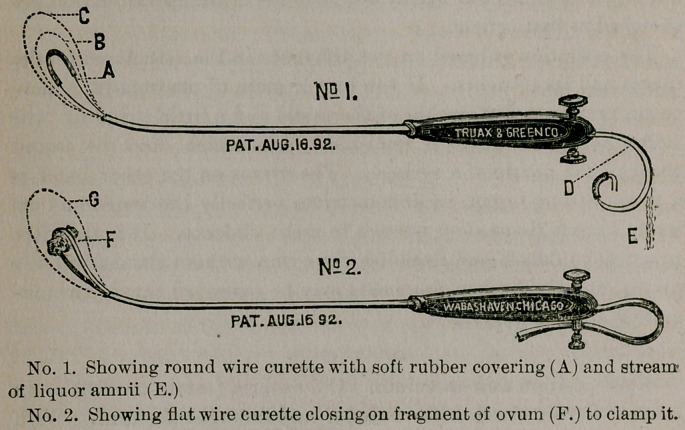# Abortion in an Hour

**Published:** 1893-06

**Authors:** Chas. H. Harris

**Affiliations:** Cedartown, Ga.


### ABORTION IN AN HOUR.

BY CHAS. H. HARRIS, M. D.,

CEDARTOWN, GA.

In the journals of last fall there appeared an article under the above caption in which was the statement that "any case of abortion in which an operation was admissible may be brought to a finish in an hour." It has been received with such marked favor showing a want of "something better" in these cases, that it may not be amiss to fill out briefly the skeleton of the operation roughly sketched in that article.

The operation is based on the difference in the structures of the uterus and its contents. If you bray a piece of placenta in a porcelain mortar, a few strokes of the pestle and a little grinding will suffice to reduce it to a semi-fluid mass which may be forced through the nozzle of a syringe. The uterus on the other hand is a tough, strong organ, its endometrium perfectly lubricated and its white fibrous tissue stout enough to resist violence. It is therefore practical to detach and demolish the ovum without damage to the uterus, and the broken fragments may be extracted through a moderately dilated cervix.

### OPERATION.

Sims' position and speculum. Chloroform (rarely needed) as in labor. Cervix fixed with volsella and brought forward. With Sims' dilator or other suitable instrument ascertain the size and dilating qualities of the cervix. Dilate to the point of divulsion. Now manipulate the rubber loop of snare No. 1 (A) to suit the size of the canal, as large as will pass with moderate pressure and use it as a probe to define the boundaries of the placenta. While yet in the uterus use this instrument to detach the placenta from its anchorage. Do this rapidly and *thoroughly*, continuing the curetting awhile after you think the work well done. The success of the operation depends largely on the thoroughness of the curetting. Snaring will be a failure without it. Not only this but it will greatly lessen the bleeding. Scraping a mortar with a thin spatula illustrates the manner of using the curette. With the same instrument while yet in the uterus, now demolish the ovum. Contract the loop to its smallest size, tighten the thumb screw and churn the uterus in all directions, using only sufficient force to plough through and break the ovum. The soft rubber covering to the wire will effectually protect the uterus (No. 1, A). The moment the membranes are ruptured the liquor amnii will pass through the tunnel of the curette and trickle down the wires in a small stream (No. 1, E). Detached and reduced in size by the churning, the freed mass now becomes a compress upon which the

No. 1. Showing round wire curette with soft rubber covering (A) and stream of liquor amnii (E.)

No. 2. Showing flat wire curette closing on fragment of ovum (F.) to clamp it.

uterus strongly contracts and forces towards the os. Snaring now begins. It is best done with large broad loops which may be sprung in the uterus or the instrument withdrawn and the loop manipulated to suit. Sweep the enlarged loop under the mass and push it up to the superior wall giving the instrument a twisting bi-lateral movement to make it encircle the ovum. Now contract the loop by sliding the staff off the curette up on the wires (No. 2, F) and tighten the thumb screw. The mass is thus clamped in the embrace of a tourniquet from which there is no escape except by division. Now pull gently and steadily with forceps movement. It is easy to understand how the ovum becomes at once a perfect tampon and the best of dilators and the uterus is aroused to activity through reflex agencies. Should the wire cut through repeat the snaring until the mass is chopped into fragments of easy extraction.

Snare No. 2, with flat watch spring wire, will catch small fragments that might escape from the round wire loop. It is intended rather as a scrape to remove residual placenta and detritus of the ovum, to put the finishing touches to the operation. Its loop is now passed into the uterus and enlarged as much as it will carry. Carefully scrape the entire wall of the uterus and pass the loop to and fro into and out of the uterus several times until the fragments cease to appear. The toilet of the organ is now completed by swabbing with a styptic antiseptic. (Tr. iodine ʒij, acid carbol. ʒi. Mix and add, when used, hot water, ℥iv.) It promptly assists the hemorrhage if you have done your work well.

This is the operation in the main. Exceptional cases will require changes to suit them. It has occurred to nearly every doctor to encounter cases with ovum loose or movable in the uterus. Such cases need only snaring. They would relieve themselves in due time, but the woman would like you better and pay you more if you pass a snare either with or without a speculum and relieve her at once. This work is easiest and best done in the office. When the "walking cases" are taught to come to your office a great advantage will be gained towards capturing this caravan of practice as yet untouched. It will put a stop to gossipping, which makes the women avoid us. With the present facilities for transportation and smooth running elevators there is no reason why it should be. Should he please a few women and prove himself worthy of their confidence, other good women will catch on and bring him more work. There are cases of abortion in which there is danger of tetanus or shock from any operation. Such cases may bear curetting which may be thoroughly and almost painlessly done with the rubber curette. The women describe the sensation as "not positive pain but as if something were tearing loose." After thorough curetting and rupturing the membranes, in a few hours spontaneous expulsion with little bleeding will take place. If not under rest and anodynes the parts will be in favorable condition for snaring the next day. There are exceptional cases. Ordinarily it is practicable and best to do both curetting and snaring at one sitting and in less than an hour.

I have done this operation in the dorsal position with Nott's speculum* in a half hour and my patient did not keep her bed. "Keep your bed, madam" is the regulation order in these cases. Never was a greater mistake. The women know better and except for cause such as hemorrhage, pelvic tenderness or fever they go about their avocations while aborting. Facts of daily occurrence prove abortion *per se* to be more troublesome than dangerous. Associated however with dysentery, enteritis, the fevers, diabetes, Bright's disease or cardiac trouble, it becomes a matter of grave import. Who has not lost a case with one of these complications? When this operation is understood the doctor will not wait for the slow approach of asthenia before he acts, but, taking time by the forelock and emptying the uterus early, he will eliminate this factor from his case.

## Figures and Tables

**No. 1. No. 2. f1:**